# Adults’ spatial scaling: evidence from the haptic domain

**DOI:** 10.1007/s10339-019-00920-3

**Published:** 2019-05-03

**Authors:** Magdalena Szubielska, Wenke Möhring

**Affiliations:** 1 Institute of Psychology, The John Paul II Catholic University of Lublin, Al. Racławickie 14, 20-950 Lublin, Poland; 2 Faculty of Psychology, University of Basel, Missionsstrasse 60/62, 4055 Basel, Switzerland

**Keywords:** Spatial cognition, Spatial scaling, Mapping task, Haptic perception, Non-informative vision

## Abstract

The current study investigated adults’ spatial-scaling abilities using a haptic localization task. As a first aim, we examined the strategies used to solve this haptic task. Secondly, we explored whether irrelevant visual information influenced adults’ spatial-scaling performance. Thirty-two adults were asked to locate targets as presented in maps on a larger or same-sized referent space. Maps varied in size in accordance with different scaling factors (1:4, 1:2, 1:1), whereas the referent space was constant in size throughout the experimental session. The availability of irrelevant, non-informative vision was manipulated by blindfolding half of the participants prior to the experiment (condition without non-informative vision), whereas the other half were able to see their surroundings with the stimuli being hidden behind a curtain (condition with non-informative vision). Analyses with absolute errors (after correcting for reversal errors) as the dependent variable revealed a significant interaction of the scaling factor and non-informative vision condition. Adults in the blindfolded condition showed constant errors and response times irrespective of scaling factor. Such a response pattern indicates the usage of relative strategies. Adults in the curtain condition showed a linear increase in errors with higher scaling factors, whereas their response times remained constant. This pattern of results supports the usage of absolute strategies or mental transformation strategies. Overall, our results indicate different scaling strategies depending on the availability of non-informative vision, highlighting the strong influence of (even irrelevant) vision on adults’ haptic processing.

## Introduction

Spatial scaling involves mapping distances between different-sized spaces (Frick and Newcombe 2012). It is an important ability that factors into several activities in our daily lives when, for example, navigating a new city and using distance information as provided on a small-scale map. Spatial scaling is a crucial skill not only for daily business but also for children’s academic achievement. Several studies have indicated relations between spatial scaling and science achievement in general (Hodgkiss et al. 2018), and mathematical reasoning in particular (Boyer and Levine 2012; Frick 2018; Möhring et al. 2015, 2018).

Although research on the effects of different-scaled objects has been conducted for more than 40 years (e.g. Bundesen and Larsen 1975; Larsen and Bundesen 1978), only a few studies have so far explored the underlying processes of adults’ spatial scaling (e.g. Möhring et al. 2014, 2016). In a typical spatial-scaling task, participants are presented with a small-scale space containing a target object (e.g. a map showing a target location) and asked to translate the same information onto a larger referent space (i.e. pointing to the same target location in the referent space). There are several ways of solving such a spatial-scaling task. One possibility refers to the usage of absolute distances. With such a strategy, the distance in the small-scale space would be encoded in an absolute way and identically mapped onto the other space without taking the size differences between the spaces into account. Such a strategy works well when spaces are very similar in size but becomes increasingly erroneous the more the spaces differ in size. As a result, errors would increase the more the two spaces differ in size (i.e. with a higher magnitude of scale translation), resulting in a linear increase in errors with higher-scale transformations. By contrast, such a simple mapping in an absolute strategy would not affect participants’ response times (RTs).

A second strategy to solve a spatial-scaling task includes the usage of relative distances (Huttenlocher et al. 1999). With such a strategy, a target would be encoded as, for example, being half the distance between two borders and be located in a different-sized space using this relational information. Such a relative strategy does not depend on absolute sizes and thus participants’ RTs and errors would not vary with the magnitude of scale translations.

A third way of solving spatial-scaling tasks refers to the usage of mental transformation strategies. Using this strategy, participants may mentally expand or shrink the size of a space in order to map it to the size of another space. Such a zooming strategy has been thought of as a magnifying glass (cf. Vasilyeva and Huttenlocher 2004) and would result in a linear increase in RTs and errors with higher scaling magnitude. These linear increases in participants’ responses occur because larger transformations (with higher scaling magnitude) take more time and entail more errors. This expectation was based on various studies in mental rotation research (e.g. Kosslyn et al. 1990; Marmor 1977; Shepard and Metzler 1971). This latter line of research has repeatedly found that mentally rotating an object for a larger angle was linked to longer RTs and higher errors than doing so for a smaller angle, similar to rotations in the physical world.

Several spatial-scaling studies have indicated that RTs and errors increased linearly with an increasing scaling factor, suggesting that adults do indeed use mental transformation strategies (Möhring et al. 2014, 2016). The majority of these studies have used localization tasks, in which adults were presented with small-scale maps showing target locations and asked to locate these targets at the same spot in another space (e.g. Frick and Newcombe 2012; Möhring et al. 2014, 2015). Some studies have also used discrimination tasks, in which participants were asked to discriminate different-sized distances (Gilligan et al. 2018; Möhring et al. 2016). Importantly, all of these previous studies tested spatial scaling exclusively for the visual domain. Consequently, it remains unclear whether mechanisms are identical when spatial information is scaled in another perceptual domain. The current study aimed to fill this gap by investigating how adults scale spatial information presented in the haptic domain and explores whether similar underlying processes are used for scaling. Answering this question is crucial because spatial information is not only perceived by the visual sense alone but also by our haptic sense. That is, our haptic sense provides crucial information about objects in our peripersonal space (i.e. the space surrounding our body), such as information about size, shape, structure, location, or the orientation of objects.

There are several reasons to believe that the processes for spatial scaling in the visual and haptic modalities resemble each other. For example, in experiments comparing participants’ tactile and visual recognition of simple maps, it was found that absolute pointing errors and RTs were nearly identical for haptic and visual learning (Giudice et al. 2011). Moreover, size changes impaired the recognition performance of 2D patterns and 3D objects for both the haptic and visual modalities (Craddock and Lawson 2009a, b; Srinivas et al. 1997; Szubielska 2015). For example, in one of these studies, it was found that sighted, but blindfolded participants recognized 2D patterns of a different size less accurately than same-sized patterns (Srinivas et al. 1997). Another study indicated that blindfolded participants showed longer RTs when recognizing different-sized 2D figures as opposed to same-sized figures, even though accuracy was not affected (Szubielska 2015). A similarly impaired performance was found when presenting participants with different-sized 3D objects that they could not see. In the haptic (but also in the visual) condition, participants showed larger errors and RTs when recognizing an object of a different size as opposed to a same-sized object (Craddock and Lawson 2009a, b). Overall, it seems that the visual and haptic modalities are deeply interconnected in object processing (Lacey and Sathian 2014), and similar processes may underlie spatial scaling for both modalities. However, to our knowledge, research using tactile stimuli has not yet systematically manipulated scaling magnitude in a haptic spatial-scaling task. Thus, at present, it remains unknown whether participants use mental transformation strategies in the haptic domain similar to those in the visual domain.

A different set of studies has shown that haptic perception can be influenced by information from another modality even when this information is irrelevant to the task (when being “non-informative”, e.g. Chan and Newell 2013). For example, it was found that such irrelevant, non-informative vision improved tactile acuity (Eads et al. 2015), haptic spatial perception and processing (Newport et al. 2002; Volcic et al. 2008; Zuidhoek et al. 2004), as well as haptic spatial memory (Pasqualotto et al. 2013a). Visual, irrelevant information may influence haptic perception and cognition because vision is seen as the “gold standard” in providing spatially precise information, to which other modalities subordinate (Myklebust 1964; Pasqualotto et al. 2013b). In general, it is assumed that non-informative vision increases the chances of creating a mental image, in which haptic information is then integrated (e.g. Pasqualotto and Newell 2007; Pasqualotto and Proulx 2012; Postma et al. 2008; Zuidhoek et al. 2004). Furthermore, there is growing evidence suggesting that non-informative vision may cause a shift from an egocentric reference frame (i.e. encoding spatial information towards one’s own body) to an allocentric reference frame (i.e. encoding spatial information towards the environment, e.g. Volcic et al. 2008; Zuidhoek et al. 2004). An environment-centred (allocentric) encoding is more invariant to changes than a body-centred (egocentric) encoding which ultimately facilitates accurate haptic perception (cf. Marmor and Zaback 1976; Millar 1976; Pasqualotto and Proulx 2012; Pasqualotto et al. 2013a; Postma et al. 2008; Zuidhoek et al. 2004). Considering these findings, it may also be the case that non-informative vision affects adults’ spatial-scaling ability in a haptic task. However, previous experiments on related research topics have either prevented non-informative vision by blindfolding their sighted participants (Craddock and Lawson 2009b; Szubielska 2015) or enabled non-informative visual input by using an opaque screen, behind which the stimuli were positioned (Craddock and Lawson 2009a). Therefore, for the first time in the current study, we combined these two conditions and manipulated whether adults received irrelevant, non-informative vision (by using a curtain) or not (by blindfolding them). In so doing, our aim was to investigate any systematic effects of non-informative vision on adults’ spatial scaling.

In the present study, we used a modified, haptic version of a localization task in analogy to the task used by Möhring et al. (2014). The aims of the study were twofold. Firstly, we wanted to determine whether adults used mental transformation strategies in a haptic spatial-scaling task similar to that seen in results in the visual domain (Möhring et al. 2014, 2016). If participants used mental transformation strategies for spatial scaling, we expected a linear increase in errors and RTs with an increasing scaling factor. Secondly, we explored whether non-informative vision had a beneficial effect on adults’ performance in the spatial-scaling task and expected participants in the curtain condition (with non-informative vision) to show more accurate spatial-scaling performance as compared to participants in the blindfolded condition (without non-informative vision).

## Methods

### Participants

Thirty-two adults aged between 19 and 48 years participated in the current study (16 females, *M*_age_ = 22.56, SD = 6.27, 31 right handed). All participants had normal or corrected-to-normal vision. Participants were predominantly university students.

### Stimuli

**Fig. 1:**
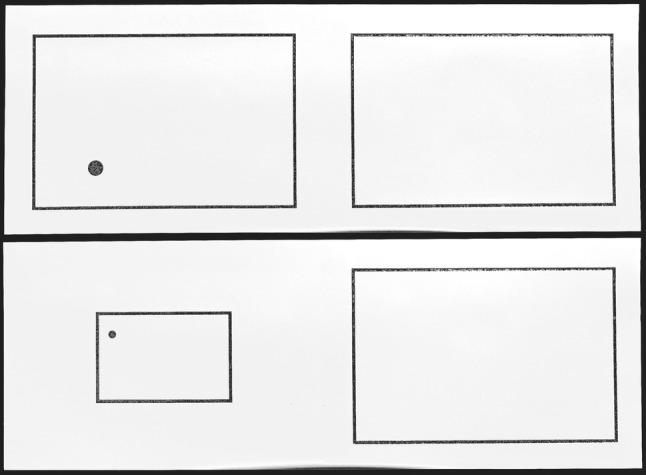
Examples of two boards with embossed graphics. Participants were presented with a map (on the left) and a referent space (on the right). The dark grey colour for borders and the target represents the convexity

Participants were presented with 148.5 mm high × 420.0 mm wide boards with embossed graphics. Each board contained two rectangular spaces: a small space that was presented on the left side and a larger space presented on the right side (see Fig. 1). In line with the study of Möhring et al. (2014), participants were first allowed to tactically encode the convex borders of the left space that contained a convex target (i.e. a dot representing a ball). Then they were asked to indicate a similar location for this target in the empty right space which was again perceptible by its convex borders. In common with previous studies investigating spatial scaling (e.g. Huttenlocher et al. 1999; Möhring et al. 2014; Vasilyeva and Huttenlocher 2004), we refer to the left space as being the “map” and the right space as being the “referent space”. Whereas the referent space was constant in size throughout the test trials (110.0 mm high × 170.0 mm wide), maps varied in size with the goal to manipulate the scaling factor. Maps ranged from 27.5 mm × 42.5 mm (scaling factor: 1:4), to 55.0 mm × 85.0 mm (scaling factor: 1:2), and to 110.0 mm × 170.0 mm (scaling factor: 1:1).^1^ Maps were centred at the same location throughout the testing session. For each of the three scaling factors (1:4, 1:2, 1:1), participants were presented with seven different target positions on the map. These target positions varied in two dimensions (see Table 1). The diameter of the targets ranged from 2.5 mm (scaling factor: 1:4) to 10 mm (scaling factor: 1:1). Participants used a 1-cm large disc to indicate the target position on the referent space. For the task instructions, an additional board with two empty spaces was used.

**Table 1:** Correct target locations (in mm) on the referent space

Target location	*X*-coordinate	*Y*-coordinate
L1	17.5	85
L2	40	25
L3	62.5	85
M	85	55
R3	107.5	25
R2	130	85
R1	152.5	25

L1 = first from the left; L2 = second from the left; L3 = third from the left; M = in the middle of the field; R3: third from the right; R2 = second from the right; R1 = first from the right

### Design and procedure

The scaling factor (1:4, 1:2, 1:1) and target location (7, cf. Table 1) were combined, amounting to a total of 21 trials. These trials were presented in a random order. Participants were randomly assigned to one of the two conditions, in which participants were either presented with irrelevant, non-informative vision (curtain condition) or not (blindfolded condition). Individuals examined in the curtain condition (*n* = 16, 8 females) were asked to put their hands behind a vertical black textile curtain. The boards with embossed graphics were then placed behind the vertical curtain to prevent vision (for similar procedures, cf. Craddock and Lawson 2009a; Newell et al. 2005; Pasqualotto et al. 2013a). Participants in the blindfolded condition (*n* = 16, 8 females) were blindfolded prior to the study and were identically presented with the embossed graphics. Participants in both conditions were allowed to freely explore the embossed graphics using both hands and were not instructed with respect to a particular exploration strategy.

The study was conducted in a single session and took approximately 25 min. Participants were tested individually in a quiet room while sitting at a table. The boards with embossed graphics and a disc were placed on the table (with the disc being presented next to the bottom right corner of the board). Each testing session began with a practice trial, in which the experimenter explained the task. Adults were instructed to encode the location of the target in the map and to place the disc at the same spot on the referent space. Participants were asked to perform the task as accurately and quickly as possible. Each test trial began when the experimenter placed a board on the table and gave a signal (by saying “start”) and was finished when the participant placed the disc on the board and signalled that this decision was final (by saying “ready”).

The experimenter measured response locations in *x*- and *y*-coordinates using a ruler (in mm) after each of the participant’s answers. Absolute errors were computed as the distance between the correct target position and each participant’s response. RTs (in seconds) were measured using a stopwatch (from the moment the participant touched the board to the moment he or she indicated that this decision was final).

### Statistical analyses

Analyses were performed using IBM SPSS 25.0. In a first step, we investigated the direction of participants’ errors by computing signed errors. These signed errors indicate (imagined) reference points that adults were using when locating the targets and to which their answers were biased (cf. Frick and Newcombe 2012). Signed errors were defined as the deviation between the *x*-coordinates (in mm) of each participant’s answer from the *x*-coordinate of the respective target location (i.e. 17.5 mm for L1). A signed error of 0 indicates a perfectly fitting localization of the target. A positive signed error indicates that participant’s answer was located too far to the right on the referent space; a negative signed error suggests that participant’s answer was located too far to the left on the referent space (cf. Frick and Newcombe 2012). To investigate the effects of the scaling factor and the non-informative vision condition on participants’ signed errors, an ANOVA was computed with the scaling factor (1:4, 1:2, 1:1) and target location (L1, L2, L3, M, R3, R2, R1) as within-participant variables, and the non-informative vision condition (curtain and blindfolded) as the between-participants variable. In this and the following ANOVAs, Greenhouse–Geisser corrections were used whenever necessary to account for violations of the sphericity assumption. The level of significance was defined as 0.05. Significant effects in ANOVAs were followed up by post hoc comparisons using Bonferroni adjustments.

In a second step, we controlled whether the participants produced left–right reversal errors when locating the targets. In these errors, the target was placed on the opposite side on the referent space (e.g. on the right half of the referent space for a target located on the left side of the map). Such reversal errors have often been found in children (cf. Frick and Newcombe 2012; Huttenlocher et al. 1994; Möhring et al. 2014) but are less common in adults (cf. Plumert et al. 2019). A typical procedure with these reversal errors is to fold participants’ answers in the middle of the horizontal dimension and to calculate the distance between the correct target position on the referent space and the folded answer (e.g. Huttenlocher et al. 1994; Möhring et al. 2014; Plumert et al. 2019). By doing so, absolute errors (i.e. the distance between participants’ answer and the correct target location) can be viewed independently of whether they were given on the left or right side of the space. As a result, participants’ answers are less biased by such extreme errors. After correcting for these reversal errors, analyses were calculated with participants’ absolute errors as well as response times as dependent variables. More concretely, to investigate the effects of the scaling factor and non-informative vision condition on participants’ responses, we calculated two repeated measures analyses of variance (ANOVA), with scaling factor (1:4, 1:2, 1:1) as a within-participant variable, and the non-informative vision condition (curtain, blindfolded) as a between-participants variable, and participants’ absolute errors as well as response times as dependent variables. Descriptive statistics on the dependent variables (i.e. signed errors, reversal errors, absolute errors after correcting for reversal errors, and response times) are presented in Table 2.

**Table 2:** Signed errors (in millimetres), the mean number of reversal errors, absolute errors after correcting for reversal errors (in millimetres), and response times (in seconds) as a function of scaling factor (1:4, 1:2, 1:1) and non-informative vision condition

	Scaling factor		
Non-informative vision condition	1:4	1:2	1:1
	Signed errors		
Curtain	− 3.39 (2.65)	− 2.03 (2.98)	− 1.95 (2.35)
Blindfolded	− .56 (2.65)	− .09 (2.98)	3.00 (2.35)
	Reversal errors		
Curtain	.09 (.12)	.07 (.10)	.09 (.09)
Blindfolded	.10 (.12)	.06 (.10)	.05 (.08)
	Absolute errors after correcting for reversal errors		
Curtain	27.45 (9.48)	22.17 (7.98)	17.78 (6.11)
Blindfolded	18.83 (9.72)	19.28 (5.61)	18.56 (5.44)
	Response times		
Curtain	33.69 (19.19)	35.04 (20.12)	36.99 (12.94)
Blindfolded	31.47 (9.38)	33.04 (10.67)	34.50 (10.63)

Standard deviations are presented in parentheses

### Results

#### Signed errors

**Fig. 2:**
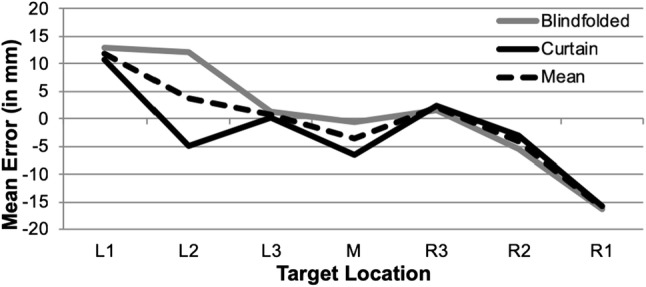
Signed errors as a function of the non-informative vision condition for different target locations on the space

To see whether participants’ responses were biased towards reference points when locating the targets, we calculated signed errors for each target location. As is seen in Fig. 2, participants located targets too far to the left on the right side of the field (i.e. as shown by negative errors), and targets on the left side of the field too far to the right (i.e. indicated by positive errors). Therefore, it seems that participants’ answers were biased towards the midpoint of the space and deviations increased with increasing distance from this midpoint. This impression was confirmed by an ANOVA with signed errors as a dependent variable. The ANOVA revealed a significant main effect of target location, *F*(4.14, 124.34) = 11.20, *p* < .001, *η*_*P*_^2^ = .27, which was best described as a linear function between target location and signed error, *F*(1, 30) = 25.85, *p* < .001, *η*_*P*_^2^ = .46 (*M*_L1_ = 11.79, SE_L1_ = 3.33 vs. *M*_L2_ = 3.06, SE_L2_ = 2.21 vs. *M*_L3_ = .66, SE_L3_ = 2.67 vs. *M*_M_ = −3.57, SE_M_ = 2.32 vs. *M*_R3_ = 1.98, SE_R3_ = 2.71 vs. *M*_R2_ = −4.18, SE_R2_ = 2.60 vs. *M*_R1_ = −16.08, SE_R1_ = 3.28). Post hoc comparisons revealed that signed errors differed significantly for the following pairs of locations: L1 and L3 (*p* = .031), L1 and M (*p* = .001), L1 and R2 (*p* = .011), L1 and R1 (*p* < .001), L2 and R1 (*p* < .001), L3 and R1 (*p* = .022), R3 and R1 (*p* < .001), and R2 and R1 (*p* = .011). A main effect of scaling factor did not reach significance, *F*(1.94, 58.06) = .68, *p* = .508, *η*_*P*_^2^ = .02. As the ANOVA yielded no significant main effect of non-informative vision, *F*(1, 30) = 1.28, *p* = .266, *η*_*P*_^2^ = .04, nor any interactions (all *F*s < 1.68, all *p*s > .155), it seems that participants of both non-informative vision conditions did not differ with respect to using reference points.

#### Reversal errors

When scanning participants’ responses, it became clear that adults sometimes produced left–right reversal errors (i.e. located a response on the wrong side of the field; for means and SDs, see Table 2). To investigate whether these errors occurred systematically, we calculated an ANOVA with scaling factor as within-participant variable and non-informative vision condition as a between-participants variable, and the number of reversal errors as the dependent variable. The ANOVA showed no significant effects (all *Fs* < 1.19, all *ps* > . 312), suggesting that these reversal errors did not vary systematically.

#### Absolute errors after correcting for reversal errors

As these reversal errors happened rarely and were not related to the variables of interest, we gave participants credit for these solutions by folding responses in the middle and looking at participants’ deviations irrespective of whether these were given on the left or right side of the space (for similar procedures, cf. Huttenlocher et al. 1994; Möhring et al. 2014, 2015; Plumert et al. 2019).

To investigate whether participants’ errors varied as a function of the scaling factor or non-informative vision condition, a repeated measures ANOVA was computed with participants’ absolute errors after correcting for reversal errors as the dependent variable. This ANOVA yielded a significant effect of scaling factor, *F*(2, 60) = 4.15, *p* = .021, *η*_*P*_^2^ = .12, which was best described by a linear function between scaling factor and errors, *F*(1, 30) = 6.61, *p* = .015, *η*_*P*_^2^ = .18 (*M*_1:1_ = 18.17, SE_1:1_ = 1.02 vs. *M*_1:2_ = 20.73, SE_1:2_ = 1.22 vs. *M*_1:4_ = 23.14, SE_1:4_ = 1.70). Post hoc comparisons revealed that participants produced more erroneous answers with a scaling factor of 1:4 than 1:1 (*p* = .046), with no differences between 1:4 and 1:2 (*p* = .561) and 1:2 and 1:1 (*p* = .243). The ANOVA revealed no significant effect of the non-informative vision condition, *F*(1, 30) = 3.93, *p* = .057, *η*_*P*_^2^ = .12, but a significant interaction of scaling factor and the non-informative vision condition, *F*(2, 60) = 3.77, *p* = .029, *η*_*P*_^2^ = .11.^2^ Follow-up comparisons showed that participants in the curtain condition produced larger errors than blindfolded participants in the condition of scaling factor 1:4 (*p* = .017), with no differences in the conditions of scaling factor 1:2 (*p* = .245), or 1:1 (*p* = .705). Moreover, post hoc comparisons revealed that it was only in the curtain condition that participants made larger errors with a scaling factor of 1:4 than 1:1 (*p* = .004; see Table 2).

Separate ANOVAs for the blindfolded and curtain condition (with identical variables) revealed a significant main effect of scaling factor for the curtain condition, *F*(2, 30) = 9.03, *p* = .001, *η*^2^ = .38, but not for the blindfolded condition, *F*(2, 30) = .04, *p* = .962, *η*^2^ = .003. This significant effect of scaling factor in the curtain condition was best explained by a linear function between scaling factor and absolute errors, *F*(1, 15) = 13.19, *p* = .002, *η*^2^ = .47, indicating that errors increased linearly with increasing scaling factors in the curtain condition (cf. Table 2), but remained constant in the blindfolded condition (for means and SDs, see Table 2). Post hoc comparisons showed that participants in the curtain condition produced significantly larger errors in spaces with a scaling factor of 1:4 than in spaces with a scaling factor of 1:1 (*p* = .017). All other post hoc comparisons were non-significant (all *p*s > .05).

#### Response times

An ANOVA with participants’ RTs as dependent variable, scaling factor as a within-participant variable, and non-informative vision condition as between-participants variable showed no significant effects (all *Fs* < 2.39, all *ps* > .101).

## Discussion

The present study investigated adults’ spatial scaling for the haptic domain and tested how non-informative vision affected adults’ performance in a haptic spatial-scaling task. Our results suggested several similarities, but also differences, between participants of the two non-informative vision conditions (blindfolded vs. curtain). For example, it was found that both groups exhibited similar directional errors. Participants’ answers were biased towards the middle, indicating that adults perceived the space as a whole and answers were gravitating towards the midpoint (cf. the category adjustment model, Huttenlocher et al. 1991). Such a pattern has typically been found for children under the age of 10 years (Huttenlocher et al. 1994), whereas adults and older children presented with visual information would typically subdivide the space into two halves and show answers biased towards the centre of each half (cf. Plumert et al. 2019). Given that adults seem to fall back on a more basic encoding and locating scheme in our haptic task, it seems that tactual encoding can be challenging even for adults (with or without irrelevant visual information).

In addition to such similarities between the conditions, our results also indicated differences. That is, depending on whether non-informative vision was available or not, adults differed with respect to their spatial-scaling strategies. Blindfolded participants who did not receive any irrelevant visual cues showed constant absolute errors and RTs across the scaling factors (cf. Huttenlocher et al. 1999). Interpretation of this non-varying, constant response pattern can be difficult. However, in the previous literature on spatial-scaling strategies (Frick and Newcombe, 2012; Gilligan et al. 2018; Möhring et al. 2014, 2016; Plumert et al. 2019) this pattern is discussed as indicating the usage of relative distances. With this kind of strategy, participants may have encoded the position of a target in relation to other landmarks (e.g. being one-third of the distance between the left and right borders). On the contrary, it was found that adults who obtained non-informative vision (in the curtain condition) produced errors that increased linearly with higher scaling factor, whereas RTs remained constant. Such a response pattern may suggest the usage of absolute distances. That is, adults may have encoded the distance on the map in an absolute way and mapped a similar distance onto the referent space (irrespective of whether the size of the map changed).

When looking at participants’ RTs, it became obvious that participants took on average more than 30 s to place the target on the referent space. This large amount of time differs from the findings in previous localization tasks in the visual domain (cf. Möhring et al. 2014). In this study by Möhring and colleagues, adults responded more quickly with an average RT of approximately 1 s to place the target (measured by means of a touch screen). Together with the directional errors mentioned above, these large RTs in the haptic version of the spatial-scaling task add to the impression that perceiving stimuli only by touch can be demanding for adults. Furthermore, it is possible that these large RTs may have masked possible increases in RTs with higher scaling factors. Whereas in the visual domain, participants can perceive a spatial layout simultaneously (or at least within a very short time), in the current study, participants had to sequentially explore the tactile spatial layout before being able to give their answer in the referent space. Explorating the map haptically and identifying the target will take longer for larger maps (as for example in the case of scaling factor 1:1) than for smaller maps (as for example in the case of scaling factor 1:4). Following this line of argumentation, exploration would then result in decreasing RTs with increasing scaling factor (*lower* RTs for scaling factor 1:4 than scaling factor 1:1). Based on the previous literature on spatial scaling, adults seem to use mental transformation strategies which were inferred from a linear increase in errors and RTs with higher scaling factors (*higher* RTs for scaling factor 1:4 than 1:1, Möhring et al. 2014). If participants in our study have used such mental transformation strategies, this linear increase in RTs (from using mental transformations) would be pitted against decreasing exploration times. These two opposing effects would then cancel each other out which may explain why RTs in the present study remained constant across scaling factors. Therefore, given that RTs in our study may have been differentially affected by exploration and scaling factor, it could also be the case that the linear increase in adults’ errors in the curtain condition (i.e. with non-informative vision) indicated the usage of mental transformation strategies (cf. Vasilyeva and Huttenlocher 2004) rather than mapping absolute distances. This impression was supported by the spontaneous comments of some participants demonstrating that they realized that the maps were sometimes same sized and sometimes not and that they had to enlarge the distance accordingly in the latter case.

Previous studies have shown that non-informative vision increased the likelihood of encoding targets within an allocentric reference frame, whereas adults without any visual information tended to rely on an egocentric reference frame (cf. Newport et al. 2002; Volcic et al. 2008; Zuidhoek et al. 2004). Within an egocentric reference frame, targets are encoded with respect to their own body, and thus, encoding is more strongly interfered with by changes in body postures or hand locations. Within an allocentric reference frame, targets are encoded with respect to the environment which is typically more robust and invariant than one’s own body. Indeed, the previous studies have indicated that this allocentric encoding resulted in more accurate performance (e.g. Postma et al. 2008). In addition to non-informative vision, there are also other conditions that increased the probability of allocentric encoding. For example, adults who turned their head towards the referent stimulus without receiving additional visual information were more likely to be using an allocentric reference frame (Zuidhoek et al. 2004). Furthermore, allocentric encoding was enhanced when implementing a delay between encoding a referent and mapping this information on a test stimulus (e.g. Milner et al. 1999; Rossetti and Regnier 1995; Zuidhoek et al. 2003). Researchers proposed that these three experimental manipulations (i.e. providing non-informative vision, performing head turns, implementing delays) may increase the tendency that a visual image is generated and the haptic input will be integrated into this visual image (e.g. Pasqualotto and Newell 2007; Pasqualotto and Proulx 2012; Postma et al. 2008; Zuidhoek et al. 2004). In line with this conclusion, Postma and colleagues (2008) stated that during the delay “the haptic input might be transformed into an allocentric representation which could be critically dependent on visual imagery ability” (p. 66).

Therefore, it may be the case that allocentric encoding is more closely related to participants’ ability to mentally generate a visual image and thus, to the analogue mental representations that are functionally equivalent to our physical surroundings (e.g. Kosslyn 1975). In other words, non-informative vision may increase the probability of mentally imagining the space perceived by touch in the form of a quasi-map (cf. Ishikawa and Montello 2006) which is then enlarged or shrunk using mental imagery, similar to the zooming strategy found in visual spatial-scaling tasks (Möhring et al. 2014, 2016). With the data at hand, however, we cannot pinpoint whether participants have used mental transformation strategies or erroneous absolute strategies when scaling distances in this non-informative vision condition. In the case of the individuals who were blindfolded and did not receive any visual information, our data suggested the usage of relational distances. Here, it may be the case that these participants have focused on their body as an available landmark which would be in line with an egocentric reference frame. While these conclusions remain speculative, it seems safe to conclude that our study revealed different scaling strategies depending on whether non-informative vision was available or not. Future studies should provide further evidence about the use of different strategies and the relations to particular reference frames in these conditions.

Contrary to our predictions, non-informative vision did not facilitate spatial-scaling ability but actually reduced participants’ accuracy—at least in the condition with the largest scaling factor. This finding contradicts the previous studies, showing that non-informative vision enhanced adults’ spatial cognition in several haptic tasks (e.g. Newport et al. 2002; Pasqualotto et al. 2013a; Volcic et al. 2008; Zuidhoek et al. 2004). Even though comparisons between our study and these previous studies may be difficult as none of these studies have examined adults’ ability to scale different-sized distances in the haptic domain, a few discrepancies between the research procedures may account for these contradictory results. For example, former studies have often manipulated non-informative vision using a horizontal curtain (Newport et al. 2002; Volcic et al. 2008; Zuidhoek et al. 2004), whereas we have used a vertical curtain. A horizontal curtain enables a larger visual field which might be a crucial aspect for encoding targets in an allocentric reference frame. However, this explanation seems improbable considering that there is another study that also used a vertical curtain which did result in beneficial effects of non-informative vision (Pasqualotto et al. 2013a). But in contrast to this latter study from Pasqualotto and colleagues, we did not implement a delay between presenting the target and giving the answer which may have been an important aspect in this experimental procedure (cf. Milner et al. 1999; Rossetti and Regnier 1995; Zuidhoek et al. 2003).

The unpredicted result of a reduced scaling accuracy in the non-informative vision condition may be explained by an additional working memory load that was added by irrelevant visual information in our task (cf. Del Gatto et al. 2016). Whereas blindfolded participants were able to focus solely on the tactile information presented, participants in the curtain condition may have been distracted by additional, irrelevant information in addition to haptically encoding the stimuli. This memory load by task-irrelevant visual information might have had the largest effects in the condition with the scaling factor of 1:4 because this scaling factor required the largest mental transformations (cf. Cornoldi and Vecchi 2003). Future studies may replicate our findings and specifically test this working memory hypothesis in a non-informative vision condition in the context of scaling tasks.

The present study has strengths and limitations. We consider it a strength that sizes of spatial layouts were systematically varied in order to manipulate the scaling factor in the present study. Another strength is that, for the first time, we manipulated the availability of irrelevant, non-informative vision in a haptic task which required encoding distances of different sizes. A limitation of the present study concerns the measurement of the RTs, highlighting the need for the future studies that measure RTs and disentangle different phases of task performance such as perceiving the haptic stimuli, performing a mental operation, and locating this information physically onto the referent space (cf. Szubielska and Bałaj 2018). Secondly, we did not assess particular exploration strategies. For example, future studies may investigate whether, and how often, participants used their fingers or hands in order to measure distances, or whether they encoded the location of the borders in relation to the target. Thirdly, we did not control participants’ head position which could have been an important influencing variable for whether they were using an allocentric or an egocentric reference frame (Zuidhoek et al. 2004).

Overall, this is the first study investigating how adults mapped and scaled different-sized distances in the haptic domain. We have found that adults showed large RTs to perform the haptic spatial-scaling task, suggesting that encoding and scaling tactile distances can be challenging. Furthermore, the results indicated that depending on the availability of non-informative vision, adults seemed to prefer qualitatively different spatial-scaling strategies. Future work could disentangle how different strategies relate to the usage of different reference frames and provide additional evidence for similarities and differences on haptic, visual, and cross-modal comparisons of such spatial-scaling tasks.

## Footnotes

1 After consulting with experts in the preparation of tactile graphics for blind individuals, it was found that larger and smaller maps than the ones used in the current study are hardly interpretable by touch.

2 A similar ANOVA calculated on non-corrected errors (i.e. on absolute errors without giving credit for reversal errors) did not reveal a significant interaction between scaling factor and the non-informative vision condition, whereas the patterns of main effects remained unchanged.
